# Thyroid Nodule Surveillance in Transfusion-Dependent Thalassemia: A Comparative Ultrasonographic Study

**DOI:** 10.3390/jcm14207265

**Published:** 2025-10-15

**Authors:** Maddalena Casale, Martina Errico, Raffaella Origa, Paolo Mureddu, Francesca Allosso, Lucia Digitale Selvaggio, Graziella Grande, Claudia Di Ludovico, Raffaele Navarra, Domenico Roberti, Maria Chiara Capellupo, Silverio Perrotta, Daniela Pasquali

**Affiliations:** 1 Department of Woman, Child and General and Specialized Surgery, University of Campania L. Vanvitelli, 80138 Naples, Italy; maddalena.casale@unicampania.it (M.C.); domenico.roberti@unicampania.it (D.R.); mariachiara.capellupo@gmail.com (M.C.C.); silverio.perrotta@unicampania.it (S.P.); 2 Department of Advanced Medical and Surgical Sciences, University of Campania L. Vanvitelli, 80138 Naples, Italy; martina.errico2@studenti.unicampania.it (M.E.); francesca.allosso@unicampania.it (F.A.); lucia.digitaleselvaggio@unicampania.it (L.D.S.); graziella.grande@studenti.unicampania.it (G.G.); claudia.diludovico@studenti.unicampania.it (C.D.L.); raffaele.navarra@studenti.unicampania.it (R.N.); 3 Department of Medical Science and Public Health, University of Cagliari, 09124 Cagliari, Italy; raffaella.origa@aob.it (R.O.); paolo.mureddu@aob.it (P.M.)

**Keywords:** thyroid, nodule, thalassemia, cancer, TIRADS, fine-needle aspiration

## Abstract

**Background:** Thyroid nodules are common in the general population, and up to 15% may be malignant. Patients with transfusion-dependent thalassemia (TDT) are predisposed to endocrine complications, raising concern for thyroid malignancy. This study compared surveillance strategies between TDT patients and healthy controls (HCs). **Methods:** This cross-sectional study used thyroid ultrasonography (US) to identify and characterize thyroid nodules in patients with TDT and HCs. Nodule assessment was performed using the Thyroid Imaging Reporting and Data System and the Italian Consensus for the Classification and Reporting of Thyroid Cytology for FNAs. Rates of indicated but unperformed FNAs and confirmed thyroid cancer cases were recorded. Statistical comparisons were performed using Fisher’s exact and chi-squared tests. **Results:** A total of 156 TDT and 101 HCs underwent thyroid US. Nodules were detected in 35.2% of TDT patients and 34.6% of HCs, with no difference in prevalence. Nodules were smaller in TDT patients, but suspicious ultrasound features and cancer prevalence were similar. Furthermore, 33.3% of TDT patients vs. 4.5% of HCs did not undergo indicated FNA (*p* = 0.021). **Conclusions:** Thyroid nodule prevalence and malignancy risk were comparable in TDT patients and HCs. A higher proportion of TDT patients missed indicated FNA procedures, revealing a critical gap in surveillance. Enhanced adherence to guideline-based follow-up is needed in thalassemia care.

## 1. Introduction

Thyroid nodules are a common finding in the general population [1]. While thyroid nodules are mostly benign, the fact that a notable percentage of 10–15% are malignant underscores the importance of diagnostic assessment for appropriate management [1,2]. Ultrasound (US) is considered the gold standard for studying thyroid nodules and can help detect small or multiple nodules and regional lymphadenopathies. US is also crucial for selecting nodules that should undergo fine-needle aspiration (FNA) cytology [2]. Classification systems like the Thyroid Imaging Reporting and Data System (TIRADS) and the European Thyroid Imaging Reporting and Data System (EU-TIRADS) have been developed for ultrasound malignancy risk stratification of thyroid nodules in adults [3,4,5,6]. These systems utilize features like shape, margins, echogenic foci, and echogenicity, in addition to size, to help differentiate between benign and malignant nodules. Different studies reported high diagnostic accuracy of these systems in predicting malignancy in thyroid nodules [7]. Several variations of TIRADS exist, with the most prominent being ACR TIRADS, EU TIRADS, and K TIRADS, each with slightly different criteria and scoring systems. Each system presents strengths and weaknesses, but they help make decisions about patient management. Transfusion-dependent thalassemia (TDT) is a hereditary blood disorder characterized by deficient synthesis of globin chains, resulting in severe hemolytic anemia [8,9,10,11].

Treatment for TDT involves regular blood transfusions, which lead to iron overload in many organs, including endocrine glands [12,13,14,15,16]. Advances in medical management, including the earlier and better management of iron overload, have improved the quality of life and life expectancy for TDT patients [17]. However, despite these improvements, complications affecting various organs, including endocrine disorders, continue to be observed. In non-transfusion-dependent thalassemia (NTDT) and other haematological disorders, endocrine complications are less frequent but still higher than in the general population, with the prevalence increasing with age [18,19]. Beyond common endocrine issues like hypothyroidism, which is frequently encountered in iron-overloaded thalassemia patients, the incidence of thyroid cancer in individuals with thalassemia has been reported to be higher than in the general population [20,21]. Papillary thyroid carcinoma (PTC) is the most common type of thyroid cancer, and several cases of PTC in adult patients with β-thalassemia have been documented. A survey across 18 international centers reported that 0.41% of adults with thalassemia major had thyroid papillary and follicular carcinoma [21]. Notably, the first reported pediatric case of PTC in a patient with β-thalassemia has recently been described [22]. The potential etiology for the increased incidence of thyroid cancer in thalassemia patients has been speculated to be linked to iron overload. Iron overload may promote carcinogenesis through various mechanisms, such as oxidative damage (via the production of oxygen free radicals), activation of ribonucleotide reductase, promotion of mutagenic hydroxyl radical formation, alteration of cytokine activity, and suppression of macrophage tumoricidal action [23]. Additionally, hepatitis C virus (HCV) infection, which can be a complication in frequently transfused patients, has also been suggested as a potential carcinogenic factor.

A recent Italian study on 4631 patients with hemoglobinopathy (48% male) followed between 1970 and 2021 for a total of 161,468 person-years reported that the rate of thyroid tumors in these patients was similar to that of the general population. This study included different types of hemoglobinopathies (TDT, NTDT, and SCD), and no difference was found in the rate of oncological diseases in the different patient groups; however, the study design did not include an evaluation of the incidence and type of thyroid nodules [8].

According to the recent indications of the Italian Society of Thalassemia and Hemoglobinopathies (SITE) [24] and the Thalassemia International Federation [25], thyroid ultrasound should be performed in cases of thyroid dysfunction or clinical thyroid enlargement.

A previous study reported a thyroid nodule rate of 8% and a malignancy rate of 31% in 195 patients with TDT, and a nodule rate of 4–7% and a malignancy rate of 5% in the general population [26].

Therefore, some authors recommended annual thyroid ultrasound surveillance and a high suspicion of malignancy if at least one of the following criteria was present: irregular shape, irregular margins, microcalcifications (<1 mm, most often round calcification), and marked hypoechogenicity [21].

Classification systems like TIRADS can be used, employing ultrasound patterns such as margin, shape, echogenic foci, echogenicity, and nodule halo sign in combination with age to help differentiate benign from malignant thyroid nodules with high sensitivity and specificity. Biopsy of suspicious nodules identified during ultrasound surveillance is also recommended.

This study aimed to assess the implementation of clinical surveillance for thyroid nodules and malignancy risk in TDT patients compared to HCs, according to the TIRADS classification.

## 2. Patients and Methods

This is a subgroup analysis of a previous multicenter prospective study on the long-term risk for endocrine complications in TDT patients [15]. This study aimed to investigate the implementation of clinical surveillance for thyroid nodules, the frequency and characteristics of thyroid nodules, and the prevalence of thyroid cancer in patients with TDT compared to HCs, according to the TIRADS classification. Consecutive TDT patients undergoing thyroid ultrasound were enrolled, alongside HCs recruited from a prevention campaign. All nodules were characterized by number, size, and TIRADS classification, with cytology assessed when fine-needle aspiration (FNA) was performed. All detected thyroid nodules were systematically assessed. This assessment included documenting their number, size, and malignancy risk stratification. The TIRADS classification system was used for risk stratification and to indicate the need for FNA procedures. In all participating centers, ultrasound examinations were performed by experienced operators. For indications for FNAs and to standardize the ultrasound evaluation in all participating centers, the TIRADS classification was used by expert endocrinologists. FNA results were interpreted according to the Italian Consensus for the Classification and Reporting of Thyroid Cytology (ICCRTC). It categorizes thyroid nodules into six main groups, each associated with a different risk of malignancy and clinical management approach (TIR1: non-diagnostic, TIR2: benign, TIR3A: low-risk indeterminate, TIR3B: high-risk indeterminate, TIR4: suspicious for malignancy, and TIR5: malignant) [27].

Healthy individuals without signs or symptoms of thyroid disease, who were part of a thyroid ultrasound prevention campaign, were included as controls in the study, after informed consent was signed.

## 3. Statistical Analysis

Statistical methods were applied to address the study objectives. Descriptive statistics summarized baseline characteristics. Group comparisons used chi-squared or Fisher’s exact tests for categorical variables, and Mann–Whitney U test for ordinal or nonparametric data. Prevalence of nodules, suspicious TIRADS categories, FNA performance, and cancer rates were prespecified outcomes. Analyses were performed in R v4.5.0, with significance set at α = 0.05. Shapiro’s normality test was used to evaluate the distribution of quantitative variables to guide the choice between parametric and nonparametric tests. For comparing categorical variables between the two groups, such as the proportion of thyroid cancer and the proportion of unperformed FNAs, Pearson’s proportion chi-squared test or Fisher’s exact test was utilized. Differences in TIRADS classification between groups were compared using the Mann–Whitney U test.

### Sample Size

The minimum number of observations required to estimate a significant difference in malignancy rates between HCs and TDT patients was obtained through a power analysis for a two independent samples proportion comparison, using a significance level of 5%, a power of 80%, and the estimated rates of 5% in the general population and 31% in thalassemic patients [26]. Results of the power analysis are reported in Table 1 and showed that a minimum of 34 observations per group were required, achieving an actual power of 81.1%.

All the analyses were performed using R v4.5.0 (R Core Team (2025). _R: A Language and Environment for Statistical Computing_. R Foundation for Statistical Computing, Vienna, Austria) as software and a significance level α equal to 0.05.

## 4. Results

A total of 156 TDT patients and 101 HCs underwent thyroid US. Nodules were detected in 55 (35.2%) TDT patients and 35 (34.6%) HCs, with no difference in prevalence (*p* > 0.99). Nodules were smaller in TDT patients (9.2 mm vs. 16.1 mm, *p* = 0.003), but suspicious features (TIRADS 4–5) and cancer prevalence were similar. Notably, 33.3% of TDT patients vs. 4.5% of HCs did not undergo indicated FNA (*p* = 0.021). In the TDT group, two patients underwent thyroidectomy for nodular goiter outside the clinical care center, and it was not possible to retrieve the ultrasound characteristics of the nodules for the present analysis (Table 2).

Among the ultrasounds where nodules were identified, the average number of nodules per ultrasound was 1.00 in the HC population and 1.31 in the TDT group. The mean maximum diameter of the largest nodule found in each ultrasound was 16.09 mm in the HC group and 9.16 mm in the TDT group (*p* = 0.003). For nodules classified as moderate and highly suspicious (TIRADS 4–5), the mean maximum diameter was 19.29 mm in the HC group and 10.89 mm in the TDT group (*p* = 0.038) (Table 2).

An evaluation of the characteristics of the detected nodules using the TIRADS classification system revealed that in both groups the TIRADS 2 and TIRADS 4 categories were the most frequently observed: in the HC group, TIRADS 4 was 31.43% and TIRADS 2 was 28.57%, while in the TDT group, TIRADS 2 was 24.53% and TIRADS 4 was 22.64%, with no significant difference (*p* = 0.64) (Table 3).

Although the TDT patients developed thyroid nodules that were smaller in size, and the diameters of the largest nodule and suspicious nodules were significantly lower than those of the HCs (*p* = 0.003), no significant difference was found regarding TIRADS classification (Mann–Whitney U = 980, *p* = 0.649) and TIR classification (Mann-Whitney’ U = 90.5, *p* = 0.796) between the two study groups (Table 3).

The rate of thyroid cancer was similar in the TDT patients (4/55, 7.2%) and HCs (6/35, 17.1%) (Fisher’s exact test *p*-value = 0.178), although a significantly higher number of TDT patients did not undergo FNA even though it was indicated according to the TIRADS classification (Table 3).

**Figure 1:**
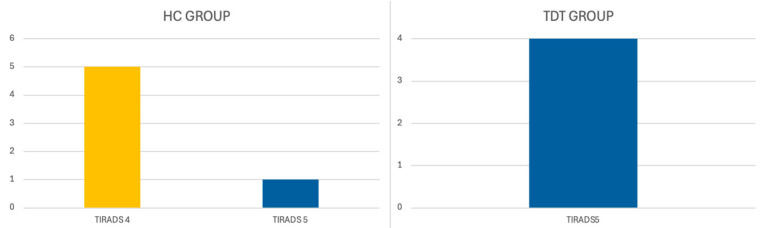
Ultrasound appearance in thyroid neoplasms.

The ultrasound appearance of thyroid neoplasms was the following: In the TDT group, histologically confirmed malignant nodules showed ultrasound features consistent with a TIRADS 5 classification. In the HC group, five histologically confirmed malignant nodules were classified as TIRADS 4, and one nodule was classified as TIRADS 5 (Figure 1).

When FNA was performed in TIRADS 4 and TIRADS 5 nodules that required FNA for dimension criteria in the HC group, the cytologic results were as follows: the eight TIRADS 4 nodules resulted in three TIR2, one TIR3A, one TIR4, and three TIR5; the four TIRADS 5 nodules resulted in two TIR2 and two TIR4.

When FNA was performed in TIRADS 4 and TIRADS 5 nodules in the TDT group, the cytologic results were as follows: the three TIRADS 4 nodules resulted in two TIR2 and one TIR3A; the seven TIRADS 5 nodules resulted in three TIR2, one TIR4, and three TIR5.

Although indicated according to TIRADS, 1 out 22 (4.5%) HCs did not undergo FNA (TIRADS 4) compared to 7 out 21 (33.3%) TDT patients (Fisher’s exact test *p*-value = 0.021). The TIRADS classification in TDT patients who missed the FNA was as follows: six nodules with TIRADS 4 and one nodule with TIRADS 5.

**Figure 2:**
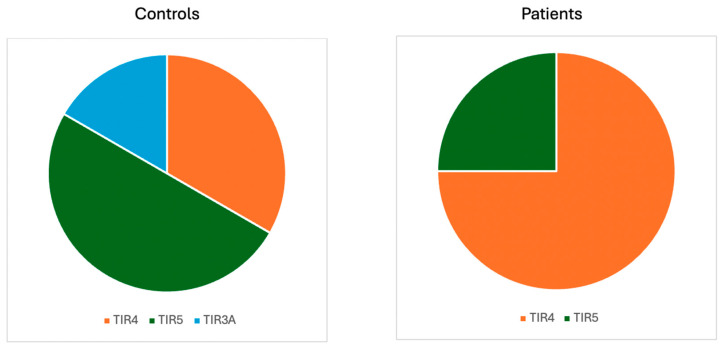
Cytological results regarding thyroid neoplasms.

Regarding the cytological evaluation of thyroid nodules in patients who underwent thyroidectomy and for whom thyroid neoplasms were histologically confirmed, the findings were as follows: in the TDT group, three nodules were classified as TIR4 and one as TIR5. In the HC group, one nodule was classified as TIR3A, two as TIR4, and three as TIR5 (Figure 2).

## 5. Discussion

This study demonstrates that thyroid nodule prevalence and malignancy risk are comparable between TDT patients and HCs, despite a smaller nodule size in TDT patients according to the clinical surveillance of thyroid nodules using thyroid ultrasonography and TIRADS classification. The most critical finding is the markedly higher rate of unperformed FNAs in TDT patients, representing a surveillance gap with potential clinical consequences.

Our results showed that thyroid nodules were present in 35.02% of the total ultrasounds performed. This prevalence rate is higher than some general population reports, such as a review in which this rate was approximately 25% [1], and significantly higher than the 6.17% reported in a study of β-thalassemia patients in Iran [28]. This variability suggests that the observed prevalence may be influenced by specific population characteristics, screening intensity, or other factors, but it underscores that thyroid nodules are a common finding in this cohort, including both thalassemia patients and controls. This finding aligns with the perspective that the occurrence of benign thyroid lesions or cancer in adult thalassemic patients is an emerging concern for physicians [29]. These observations, coupled with the results of our study, suggest that thyroid malignancy warrants focused attention in this patient population through the implementation of correct clinical surveillance. Clinical implications include the need for routine thyroid US surveillance in TDT patients, irrespective of thyroid function, and targeted interventions to improve FNA adherence.

TIRADS is an ultrasound-based classification system used to assess and stratify the malignancy risk of thyroid nodules. Its main goal is to help differentiate between benign and malignant nodules and guide decisions for further diagnostic steps like fine-needle aspiration (FNA). The TIRADS system analyzes specific ultrasound features of a nodule, including shape, margins, echogenic foci, echogenicity, and nodule halo sign, in conjunction with age, to differentiate between benign and malignant nodules. Nodules are assigned to categories (e.g., TIRADS 2 to TIRADS 5), where higher categories indicate a greater suspicion of malignancy. The most common malignancy risk classifications using TIRADS were TIRADS 2 (benign) and TIRADS 4 (moderately suspicious), each accounting for 26.14% of nodules. Magri et al. [5] and Ahmadi et al. [30] both noted that a non-negligible number of cancers would be missed if only nodules meeting FNA size criteria were biopsied. Although thyroid nodules in patients with transfusion-dependent thalassemia (TDT) are significantly smaller than those in healthy controls, this study emphasizes that nodule size alone is not a reliable indicator of malignancy risk. The smaller size of nodules in TDT patients may have influenced the decision to perform fine-needle aspiration less frequently. However, upon evaluating their ultrasound characteristics, these nodules were still considered worthy of further investigation, as they showed a similar rate of suspicious features and thyroid cancer compared to controls. It is particularly noteworthy that, in TDT patients, the nodules for which surgery was indicated following FNA already exhibited ultrasound features highly suggestive of malignancy, corresponding to a TIRADS 5 classification. This highlights the critical role of ultrasound as an early diagnostic tool for the detection of thyroid malignancies in this population.

A particularly important finding of this study was the significantly higher rate of FNA procedures that were indicated but not performed in the thalassemia group (33.33%) compared to the control group (4.55%) (*p* = 0.021). FNA is a standard diagnostic tool for evaluating suspicious thyroid nodules, and indicated biopsies are typically necessary for accurate classification and management planning [1]. The high rate of unperformed FNAs in thalassemia patients is concerning, as it could lead to delays in the definitive diagnosis of malignancy and potentially impact patient outcomes. This finding underscores a critical area for improvement in the clinical management pathway for thyroid nodules in this specific population. Reasons for this higher rate could be multifaceted, potentially including the complexity of managing multiple comorbidities in thalassemia patients, patient fatigue with numerous medical appointments and procedures, or logistical challenges in coordinating follow-up.

Given the observed prevalence of thyroid nodules and especially the challenge with completing indicated diagnostic procedures, our findings strongly support the necessity for routine thyroid ultrasound surveillance in patients with thalassemia major, even in patients without thyroid dysfunction (hypothyroidism, hyperthyroidism, and chronic autoimmune thyroiditis). This recommendation is in line with other calls for annual thyroid ultrasound surveillance and biopsy of suspicious nodules in thalassemic patients due to the emerging concern of thyroid cancer [29], and the recommendation for long-term follow-up due to potential carcinogenic effects [28]. Furthermore, the results highlight the need for improved diagnostic follow-up strategies in this group, specifically addressing the barriers that result in unperformed indicated FNAs.

This study is a cross-sectional analysis, which provides a snapshot of the prevalence at a single point in time. While it reveals important associations, it cannot establish causality or track changes over time. The lack of statistical significance regarding the comparison of cancer prevalence between groups, while potentially limited by sample size, should be interpreted cautiously and highlights the need for larger studies. Future prospective studies with longer follow-up periods, potentially multicenter collaborations, are needed to definitively assess the long-term risk of thyroid cancer in various forms of thalassemia, understand the specific impact of iron overload and other factors on tumorigenesis, and evaluate the effectiveness of different surveillance and follow-up protocols in improving patient outcomes. Investigations into the reasons for the high rate of unperformed FNAs in thalassemia patients and the development of targeted interventions to improve adherence to diagnostic recommendations are also crucial.

Strengths of the study include the prospective design and application of standardized risk classification; limitations include cross-sectional analysis, limited sample size, and lack of longitudinal outcomes. Future multicenter studies should clarify long-term thyroid cancer risk in TDT patients and evaluate strategies to overcome barriers to diagnostic follow-up.

## 6. Conclusions

TDT patients have a burden of thyroid nodules and a malignancy risk that are comparable to those of HCs, but significantly higher rates of missed FNAs. Identifying that a specific, chronically ill patient population is significantly less likely to receive the indicated standard of care for potential cancer is a highly significant clinical finding. This surveillance gap could lead to delayed diagnoses and poorer outcomes. Our conclusion highlights a need for targeted interventions to improve adherence to guideline-based follow-up in thalassemia care, which we believe is a valuable contribution to the field.

## Figures and Tables

**Table 1:** Power analysis.

	N1	N2	Actual Power	Test Assumptions				
				Power	Risk Difference	Risk Ratio	Odds Ratio	Sig.
Test for Proportion Difference ^a^	34	34	0.811	0.8	−0.260	0.161	0.117	0.05

^a^ Two-sided test using large-sample approximation. The estimation of power is based on Pearson’s proportion chi-squared test and the pooled standard deviation.

**Table 2:** Baseline characteristics of the study population by group. Continuous variables are expressed as mean (standard deviation), and categorical variables as number (percentage).

Characteristic	Group		Test	
	Healthy Controls	Patients with Thalassemia	Statistic	*p*-Value
Thyroid ultrasound performed	101	156		
Age ^1^	39.54 (16.17)	44.60 (10.30)	3283	0.006
Prevalence of thyroid nodules ^2^	35/101 (34.65%)	55 ^3^/156 (35.26%)	3.11 × 10^−30^	>0.999
Number of suspected nodules	1.00 (0.00)	1.31 (0.86)		
Maximum diameter of the largest nodule (mm) ^1^	16.09 (10.77)	9.16 (5.43)	1094	0.003
Moderate and highly suspicious nodule (TIRADS 4–TIRADS 5) diameter (mm) ^1^	19.29 (12.05)	10.89 (4.52)	190.5	0.038
Age (if thyroid nodules) ^1^	47.48 (18.13)	46.42 (10.45)	630	0.545

^1^ Wilcoxon rank sum test (Mann–Whitney test), ^2^ Pearson’s proportion chi-squared test. ^3^ In the TDT group, 2 patients underwent thyroidectomy for nodular goiter outside the clinical care center, and it was not possible to retrieve the ultrasound characteristics of the nodules for the present analysis.

**Table 3:** TIRADS and TIR classification of thyroid nodules in the study population. Continuous variables are expressed as mean (standard deviation), and categorical variables as number (percentage).

Characteristic	Group		Test	
	Healthy Controls	Patients with Thalassemia ^3^	Statistic	*p*-Value
TIRADS ^1^			980	0.649
TIRADS 1	3/35 (8.57%)	10/53 (18.87%)		
TIRADS 2	10/35 (28.57%)	13/53 (24.53%)		
TIRADS 3	8/35 (22.86%)	10/53 (18.87%)		
TIRADS 4	11/35 (31.43%)	12/53 (22.64%)		
TIRADS 5	3/35 (8.57%)	8/53 (15.09%)		
TIR ^1^			90.5	0.796
TIR1	0/16 (0.00%)	0/12 (0.00%)		
TIR2	10/16 (62.50%)	6/12 (50.00%)		
TIR3	1/16 (6.25%)	2/12 (16.67%)		
TIR4	2/16 (12.50%)	3/12 (25.00%)		
TIR5	3/16 (18.75%)	1/12 (8.33%)		
Fine-needle aspiration indicated but not performed	1/22 (4.55%)	7/21 (33.33%)		0.021
Thyroid cancer ^2^	6/35 (17.14%)	4/55 (7.27%)		0.178

^1^ Wilcoxon rank sum test (Mann–Whitney test), ^2^ Fisher’s exact test. ^3^ In the TDT group, 2 patients underwent thyroidectomy for nodular goiter outside the clinical care center, and it was not possible to retrieve the ultrasound characteristics of the nodules for the present analysis.

## Data Availability

For the original data, please contact the corresponding author (daniela.pasquali@unicampania.it).
